# Psychometric validation of a Saudi Arabian version of the sf-36v2 health survey and norm data for Saudi Arabia

**DOI:** 10.1186/s41687-020-00233-6

**Published:** 2020-08-13

**Authors:** Ahmad AboAbat, Hazem Qannam, Jakob Bue Bjorner, Mohamad Al-Tannir

**Affiliations:** 1 Consultant Rehabilitation Hospital, King Fahad Medical City, Riyadh, Kingdom of Saudi Arabia; 2 Rehabilitation Hospital, King Fahad Medical City, Riyadh, Kingdom of Saudi Arabia; 3 Optum Outcomes, Johnston, RI USA; 4 Research Center, King Fahad Medical City, PO Box 59046, Riyadh, 11525 Kingdom of Saudi Arabia

**Keywords:** Quality of life, Norms, SF-36v2, Validity, Population-based survey, Saudi Arabia

## Abstract

**Background:**

Adaptation of a patient-reported outcomes survey into a new language requires careful translation procedures as well as qualitative and quantitative psychometric testing. This study aimed to evaluate the basic psychometric properties of the new Saudi Arabian SF-36v2 and establish norm data for Saudi Arabia.

**Methods:**

Translation and adaptation of the SF-36v2 used standard methodology. Psychometric validation included two stages: 1) A qualitative study (*n* = 100) explored the components of health and health-related quality of life considered important in Saudi Arabia and evaluated the content validity of the SF-36v2 in Saudi Arabia, and 2) A quantitative study (*n* = 6166) evaluated the basic psychometric properties of the Saudi SF-36v2 and established norm data for Saudi Arabia. Comparison with US general population data (*n* = 4040) evaluated differential item function (DIF) and cross-national differences.

**Results:**

The qualitative study supported the content validity of the Saudi SF-36v2. Cognitive debriefing identified only few and minor problems. Psychometric analyses supported item convergence within scales and differentiation across scales of the SF-36v2. Scale level exploratory factor analyses did not support the typical distinction between physical health and mental health components. Internal consistency reliability was satisfactory for all scales except the social function scale (alpha = 0.67). Cross-national DIF was identified for 9 items. In the Saudi general population, the average vitality score was lower for women (− 2.71 points) compared to men. For men, older age groups scored lower on the physical function scale (− 3.31) and the physical health component (− 3.06). For women, older age groups scored lower on the role physical (− 3.72), bodily pain (− 3.66), and vitality (− 2.32) scales as well as the physical health component (− 3.52). Compared to the 2009 United States general population, and after adjusting for age, gender, and differential item function, persons in Saudi Arabia had lower average scores for the physical function (− 3.10), role physical (− 4.75), social function (− 4.23), role emotional (− 5.67), and mental health (− 4.82) scales, as well as the mental health component (− 4.57).

**Conclusion:**

This Saudi normative study of patient reported outcomes supported the validity and reliability of the new Saudi SF-36v2 and found cross-national differences with the USA.

## Background

Patients’ self-reports of health outcomes are important for measuring the impact of chronic disease, accounting for changes in health, measuring the effects of treatment, and predicting health resource utilization and thus medical expenditures. To date, most of the available patient-reported outcome (PRO) measures are in English, and few have been translated into Arabic and adapted for use in Arab countries [1]. Because the perception of health-related outcomes may differ between populations and conditions, adaptation of a questionnaire into a new language and culture requires more than just a translation. Evaluation of content validity, construct validity, and reliability as well as establishing national normative data are important steps in the translation and cultural adaptation of a PRO measure [2–9]. Despite these challenges, the literature urges investigators not to “reinvent the wheel” by developing new or ad hoc measures, but rather cross-culturally adapt an existing health and health-related quality of life (HRQOL) measure. Cross-cultural adaptation is believed to: 1) be more cost-effective; 2) enable efficient utilization of the existing body of knowledge; 3) help standardize the concept internationally; and 4) offer the opportunity for international comparative studies. A disadvantage of culture-specific instruments is that their results are not generalizable or comparable because each has its conceptual definition and choice of indicators [6].

The SF-36 is one of the most widely used PRO instruments [10]. Its validity, reliability, and responsiveness have been documented in many groups varying by age, sex, socio-economic status, geographical region, and clinical conditions [3]. In the 1990s, researchers within the well-documented International Quality of Life Assessment (IQOLA) project pioneered the adaptation of the SF-36 for use internationally [11]. The methods used in the IQOLA project, still constitute the standard for translation and validation work today. The SF-36 has been translated into more than 150 languages and adapted to different cultures [10]. Responding to the difficulties in translating various items and response choices, the IQOLA project’s investigators emphasized the importance of developing translations that are culturally appropriate to each country [2].

Published norms for the SF-36 exist in several developed countries [4, 11–21]. Norms permit evaluation of disease burden, i.e. the decrement in PRO scores relative to a general population comparison group with similar age and sex distribution [10]. Normative data can also help interpretation of treatment effects since no treatment effect can be expected to be larger than the disease burden. For Saudi Arabia, PRO population norms could help identify needs and subsequently guide health policies, legislation, and the development of strategic plans to allocate resources based on unmet needs. However, most previous work in Saudi Arabia has used the SF-36v1 or RAND-36 [22], rather than SF-36v2, and general population norms have been lacking.

Accordingly, this nationwide study aimed to explore the content validity of SF-36v2 in a Saudi Arabian context, test the validity and reliability of a new Saudi Arabic SF-36v2 translation, and collect Saudi normative SF-36v2 data. Since the SF-36v2 scoring is based on US general population norms, we also explored the difference between US and Saudi Arabian norms.

## Methods

This project was performed in 2 stages, utilizing both qualitative and quantitative methods.

The qualitative study had two objectives: 1) To explore the concepts of health and HRQOL and evaluate the content validity of the SF-36v2 as an HRQOL instrument in a Saudi Arabic setting, and 2) To perform cognitive debriefing of the Saudi SF-36v2.

Semi-structured interviews were carried out on a convenience sample of 100 participants by trained interviewers aiming to explore which domains the participants consider important components of health and HRQOL, and to ascertain concordance with the WHO definition of health as “a state of complete physical, mental, and social well-being and not merely the absence of disease or infirmity” [23]. This definition forms the conceptual basis of the SF-36 and other commonly used HRQOL measures. Participants were asked introductory questions including: “What is the meaning of health?”, “What do you think may affect a person’s health?”, and “What areas of life do you think are affected by health?” Participants were probed to elaborate on their answers until they indicated having no more ideas. Participants were then asked to evaluate the importance of domains commonly used in measuring HRQOL using a four-point scale (“very important”, “quite important”, “not quite important”, and “not at all important”). Next, participants were asked to list additional domains that were not mentioned among the listed domains. It has been suggested that indicators could be added if rated important by at least 50% of subjects [24].

Subsequently, participants engaged in a cognitive debriefing of the Saudi SF-36v2 to evaluate whether the content of the translated version was easily understood and culturally relevant within a Saudi Arabian context. After completing each item, participants were asked questions about clarity, comprehensibility, relevance, and completion feasibility using a standardized response scale. The participants were probed to elaborate on their answer, using probes such as: “Interesting, can you elaborate on that?”, “What do mean, can you explain further?”, “How important is that to you?”, “Do you want to add anything in this regard?”

The quantitative study was based on a national general population survey involving Saudis aged 15 years or older. Saudi Arabia is divided into five regions (North, South, Central, East, and West); each region is divided into sub-regions and blocks. A probability proportional sampling method was used to randomly select sub-regions, blocks, and accordingly households. Households were chosen from each block and a roster of household members (based on age and sex) was collected by a surveyor visiting the household. An adult aged 15 years or older was randomly selected to be surveyed from each household. The surveyor handed out the SF-36v2 for self-administration. Individuals were excluded if they were unable to complete the questionnaire due to language problems, communication limitations or cognitive impairments. If the selected adult was not present, our surveyors made an appointment to return. The household was counted as nonresponsive after a total of three attempted unsuccessful visits.

The Saudi population is estimated to be approximately 12,167,245 people. The study aimed to obtain 6360 completed surveys. This sample was chosen to achieve sufficient representation of all strata of the Saudi population. Based on experience with surveys in Saudi Arabia, we assumed a non-response rate of up to 40% for a target of 10,600 contacts. Of the 10,592 approached Saudi adults, 6166 participated in the study with a response rate of 61%.

The Saudi Arabian data was compared to United States (US) general population data (*n* = 4040) obtained in 2009 [25]. This general population online survey of US citizens 18 years or older has been used to generate population norms for the USA (please see [25] for details).

### Measures

SF-36v2 was administered using a new Arabic version and scored according to standard recommendations of the SF-36v2 developers [25] into eight subscales: physical function (PF), role limitations due to physical health (RP), bodily pain (BP), general health perception (GH), vitality (VT), social function (SF), role limitations due to emotional problems (RE), and mental health (MH). For each subscale: 1) items were coded so that high score indicated good health, 2) two items were weighted [26], for all other items simple category weights (1, 2, 3 …) were used, 3) the mean score was taken across items and transformed linearly to a metric from 0 to 100, 4) for norm based scoring, the scale score was transformed linearly so that the US general population has a mean of 50 and an SD of 10. Also, two overall component scores, the physical component summary (PCS) and the mental component summary (MCS) were calculated based on scoring coefficients from a principal component analysis with orthogonal rotation [27].

The translation of the SF-36v2 used the principles of good practice from the International Society of Pharmacoeconomics and Outcomes Research Task Force for Translation and Cultural Adaptation [28]. This included: 1) Forward translation by two independent professional translators, 2) Back translation by a third independent translator, 3) Reconciliation by an expert panel, and 4) Independent assessment of translation quality. The translation was subsequently evaluated by cognitive debriefing and quantitative testing, as reported in this paper.

Morbidity questions included a list of 27 self-reported health conditions (hypertension, heart disease, diabetes, arthritis, depression, etc.) and one open-ended response coded as “other”.

Sociodemographic questions included age, gender, education level, marital status, occupation, and financial status (monthly household income). Additional questions concerned smoking habits and major life events during the previous year.

### Statistical analysis

Distributions of basic demographic variables and chronic conditions were described through standard frequency tables. The analysis of scale structure relied on multi-trait analyses [29], which have been used in multiple previous studies of the SF-36. These analyses test item convergence within scales and item differentiation across scales. Item convergence within scales (sometimes called convergent validity) was evaluated by analyzing the correlation of each item with the sum of all the other items in the scale (item-own-scale correlation has been corrected for overlap, also see [30]). A correlation of 0.40 or more for all items in a scale supports item convergence within scales. Item differentiation across scales (sometimes called discriminant validity) was evaluated for each item by comparing the item’s correlation with its scale to its correlation with all other scales. Item differentiation across scales is supported if the item’s correlation with its own scale is significantly larger than its correlation with any other scale. Furthermore, we analyzed the scale correlation matrix using exploratory factor analysis as in many previous studies of the SF-36 (e.g. [31, 32]). Number of factors were evaluated by Eigen value analysis. Factors were extracted using the principal components method, followed by orthogonal rotation (Varimax). While most studies have used a two-factor solution of physical and mental health [31], analyses in some non-Western countries have suggested a three-factor structure of physical, mental, and social health [32]. For this reason, we evaluated both two- and three-factor solutions. As robustness analyses, we supplemented the Varimax rotation with an oblique rotation (Promax) and supplemented standard analyses of product-moment correlations with analyses of the polychoric correlation matrix.. Internal consistency reliability (coefficient alpha) was estimated for the subscales. Internal consistency reliability for the PCS and MCS was estimated using methods for weighted composites (see [33] page 37).

Differential item function (DIF) was evaluated for age, gender, and comparisons of Saudi Arabia and USA using logistic regression DIF tests [34]. Adopting a standard decision rule [35], evaluation of important DIF was based on statistical significance (*p* < 0.05 after Bonferroni adjustment) and magnitude in terms of increase in explained item variance (difference in pseudo R-squared [36] larger than 0.03). This criterion is slightly less conservative than a threshold of 0.035 advocated in the educational testing literature [37]. We used a standard purification strategy [34], where items with indications of DIF were excluded iteratively until a set of anchor items without DIF was identified. Then, the final DIF analyses were conducted for each item using the anchor items and the item in question. In cases of important cross-national DIF, we adjusted the cross-national comparisons using the generalized partial credit item response theory (IRT) model [38]. This model can adjust for uniform DIF (DIF with the same magnitude across score levels) by adjustment of the IRT thresholds parameters and adjust for non-uniform DIF (magnitude of DIF depends on score level) by adjustment of the IRT discrimination parameter. DIF adjustment was performed using a three step procedure: 1) We estimated item parameters for all SF-36v2 scales using the US data and the generalized partial credit IRT model [38], 2) For items with significant DIF, we re-estimated the item parameters in the Saudi data, fixing item parameters for the anchor (no-DIF) items, and 3) We performed IRT-based sum score cross-calibration to link the Saudi scale scores to the US metric [39]. After doing this for all subscales with DIF, we calculated adjusted PCS and MCS scores based on the adjusted subscales.

Comparisons between Saudi Arabia and the USA were carried out using a linear regression model, with and without controlling for differences in age and gender, and adjustment for DIF. The magnitude of differences was evaluated according to published guidelines for minimal important differences (MID) for the SF-36v2 [25]: PF: 3 points, RP: 3 points, BP: 3 points, GH: 2 points, VT: 2 points, SF: 3 points, RE: 4 points, MH: 3 points, PCS: 2 points, and MCS: 3 points. These MID values have been established using anchors such as noticeable increase in risk of mortality, job loss, or hospitalization [40].

## Results

### Health and HRQOL concepts

The characteristics of the sample (*N* = 100) used in the qualitative study are presented in Table 1.

**Table 1:** Socio-demographic Characteristics of Participants in the Qualitative Study (*n* = 100)

**Age (in years)**	Median	**30**
	STD	**14**
	Age range	**15–70**
**Gender**	Male	**79%**
	Female	**21%**
**Marital Status**	Married	**51%**
	Widowed/divorced	**3%**
	Single	**47%**
**Employment Status**	Full-time student	**11%**
	Part-time student	**2%**
	Working full-time	**72%**
	Working part-time	**5%**
	Retired	**9%**
	Homemaker	**1%**
**Education Level**	Less than high school	**19%**
	High school	**24%**
	Some College	**10%**
	College/University	**39%**
	Postgraduate	**8%**

In the qualitative study, four concepts were endorsed by 50% or more of participants as components of health: physical functioning (70% of participants), normal psychological function and feelings (66%), healthy eating habits and enjoyment of food (61%), normal social functioning (50%); 38% of participants defined absence of disease or illness and 28% being full of energy (free from pain and fatigue) as components of health (Table 2).

**Table 2:** Conceptual Domains Considered Components of Health

	%
Normal physical functioning (including general mobility, exercises, and sports)	70%
Normal psychological function and feelings (happiness, love, peace, and stress-free)	66%
Eating (healthy habits, enjoyment of food)	61%
Normal social functioning (with family and friends)	50%
Good sleep (habits, duration, quality, and uninterrupted)	49%
Normal daily activities (self-care, housework, work, schooling, etc.)	49%
Free from illness	38%
Full of energy (free from pain or fatigue)	28%
Performing religious acts (going to the mosque)	25%
Leisure functions (primarily travel and outings)	17%
Normal cognitive function	8%
Others **(**good finance, health checkup, normal weight, etc.)	7%

When presented with a list of domains commonly including in the assessment of HRQOL, concepts related to all eight SF-36 domains were assessed as “quite” or “very” important for HRQOL (Range 95% - 100%, Table 3). While the concept of being full of energy was considered as a component of health by only 28% of the sample, related concepts of “having a lot of energy” and “being free from pain” was considered a “very” or “quite” important components of HRQOL by 100% and 96% of participants, respectively. Participants also reported some additional domains that are not covered by the SF-36 as important: eating habits (72%), sleep (55%), travel (53%), and sexual function (56%) (Table 3).

**Table 3:** Importance Ratings of Health Related Quality of Life Domains

Domain	Not at all Important	Not Quite Important	Quite Important	Very Important	Important Total
Function (i.e. what a person is able to do)	0%	0%	5%	94%	99%
Having a lot of energy	0%	0%	11%	89%	100%
Ability to carry out daily activities	3%	0%	14%	83%	97%
Feelings and emotions	0%	0%	17%	83%	100%
Physically functioning	1%	0%	17%	82%	99%
Being free from pain	4%	0%	23%	73%	96%
Being a happy person	1%	0%	26%	73%	99%
Change in health	3%	0%	29%	68%	97%
Not feeling tired	5%	0%	33%	62%	95%
Social functioning	2%	0%	42%	56%	98%
Subjective health perception	2%	0%	45%	53%	98%
Additional Reported Domains^a^	Eating (habits and enjoyment)				72%
	Sexual function				56%
	Sleep				55%
	Travel (for pleasure and religious)				53%
	Environment (housing, hygiene, pollution)				39%
	Finance				25%
	Religion (relationship with God/spirituality/personal/life attitude)				22%
	Others: health status, the presence and quality of health services, relaxations and free time, success at work				19%

^a^Patient were asked “Apart from the domains mentioned above, please list all other HRQOL domains that you think are important.” results reported here as frequency of reporting of additional domains

We identified four SF-36v2 items that two or three participants (out of 100) had problems understanding: HT (*Health compared to 1 year ago*), RP4 (*Difficulty performing work due to physical health*), VT4 (*Feeling tired*), and GH3 (*Healthy as anybody*). No other problems were identified for any other item at this stage.

### Quantitative sample characteristics

Compared with the 2009 USA general population, the Saudi sample was younger (49.4% in the age range 15–29 years, with 47.3% in the age range 18–29 years vs. 22.0% in the USA), included a higher proportion of never married (43.8% vs. 26.9%), and a lower proportion of divorced/separated (3.1% vs. 15.6%). More people in the Saudi sample had received a college degree (43.2% vs. 35.0%) (Table 4). Differences in reporting of employment status precluded a detailed comparison, but 48.6% of the Saudi sample was working compared to 53.3% in the USA sample.

**Table 4:** Socio-demographic Characteristics of the Participants in the Quantiative Study

	Saudi Arabia		USA	
	Count	%	Count	%
Age group				
15–29 years^a^	2962	49.4	887	22.0
30–44 years	1695	28.3	1089	27.0
45–59 years	952	15.9	1151	28.5
60+ years	392	6.5	909	22.5
*Missing*	165		0	
Gender				
Male	2903	47.5	1940	48.1
Female	3209	52.5	2096	51.9
*Missing*	54		0	
Marital status				
Married	3111	51.0	2081	51.6
Widowed	130	2.1	239	5.9
Divorced/separated	187	3.1	630	15.6
Single/Never married	2671	43.8	1086	26.9
*Missing*	67		0	
Education				
At most high school	2575	42.8	1777	44.0
Some college or similar	840	14.0	847	21.0
College degree	2602	43.2	1412	35.0
Region				
Central	2392	38.9		
Eastern	783	12.7		
Western	2330	37.9		
Northern	126	2.1		
Southern	513	8.4		
*Missing*	22			

^a^US sample 18-29

Data on self-reported health conditions (Table 5) also showed noticeable differences between Saudi Arabia and the USA. In Saudi Arabia, the most prevalent conditions were: trouble seeing (26.1%), back problems (18.0%), anemia (15.8%), and allergies (15.3%). Trouble seeing was much less frequently reported in the USA (12.1%), but several other conditions were considerably more prevalent in the USA: allergies (47.9%), hypertension (32%), arthritis (26.4%), anxiety (17.2%), and depression (14.1%).

**Table 5:** Self-reported Health Conditions among Respondents in Saudi Arabia and the USA

	Saudi Arabia (*n* = 6088)		USA (*n* = 4400)	
	Count	Percentage	Count	Percentage
Trouble seeing	1591	26.1	533	12.1
Back problems	1098	18.0	948	21.6
Anemia	960	15.8	475	10.8
Allergies (chronic or seasonal)	932	15.3	2105	47.9
Obesity	678	11.1	782	17.8
Sleep disorders, insomnia, sleep apnea	668	11.0	438	10.0
Hypertension	624	10.2	1410	32.0
Migraine/Severe headaches	614	10.1	838	19.0
Irritable Bowel Syndrome	571	9.4	352	8.0
Ulcer or stomach disease	536	8.8	242	5.5
Diabetes	523	8.6	548	12.5
Asthma	447	7.3	415	9.4
Chronic fatigue	389	6.4	180	4.1
Gastro esophageal reflux disease	373	6.1	632	14.4
Skin conditions	312	5.1	406	9.2
Heart disease	303	5.0	527	12.0
Arthritis or chronic joint problems	297	4.9	1161	26.4
Emphysema, chronic bronchitis, COPD	247	4.1	258	5.9
Trouble hearing	181	3.0	432	9.8
Kidney disease	173	2.8	92	2.1
Cancer	119	2.0	239	5.4
Anxiety (clinical)	116	1.9	758	17.2
Depression (clinical)	111	1.8	620	14.1
Stroke	95	1.6	117	2.7
Liver disease	53	0.9	112	2.6
Limited Use of arm(s) or leg(s)	53	0.9	397	9.0

### Item convergence within scales, item differentiation across scales

Table 6 presents results of analyses of item convergence within scales and differentiation across scales in the Saudi sample. The numbers in bold show each item’s correlation with the sum of all the other items in its own scale (item-own-scale correlations). All items satisfied the standard criterion of item convergence within scales (≥0.40). For all items except one, the item-own-scale correlation was higher than the correlation with any other scale, thus supporting item differentiation across scales. One item, SF01 (“*During the past 4 weeks, to what extent has your physical health or emotional problems interfered with your normal social activities with family, friends, neighbors, or groups?*”), showed a higher correlation with the pain scale than with the other item in its own scale.

**Table 6:** Item-scale Correlations for the SF-36v2 – Saudi Arabia

		Scale								
Domain	Item	PF	RP	BP		GH	VT	SF	RE	MH
PF	PF01	**0.42**	0.38	0.36		0.36	0.39	0.28	0.26	0.18
	PF02	**0.77**	0.45	0.28		0.16	0.16	0.27	0.33	0.19
	PF03	**0.79**	0.45	0.25		0.11	0.13	0.27	0.33	0.20
	PF04	**0.66**	0.45	0.38		0.32	0.36	0.31	0.31	0.18
	PF05	**0.78**	0.45	0.29		0.22	0.23	0.28	0.33	0.13
	PF06	**0.76**	0.44	0.31		0.18	0.22	0.28	0.30	0.16
	PF07	**0.72**	0.46	0.31		0.23	0.24	0.30	0.31	0.25
	PF08	**0.78**	0.45	0.25		0.13	0.15	0.29	0.32	0.21
	PF09	**0.75**	0.42	0.23		0.10	0.13	0.28	0.31	0.18
	PF10	**0.71**	0.39	0.17		0.08	0.09	0.23	0.30	0.16
RP	RP01	0.51	**0.78**	0.46		0.34	0.34	0.47	0.56	0.26
	RP02	0.51	**0.84**	0.47		0.30	0.36	0.47	0.60	0.30
	RP03	0.50	**0.81**	0.47		0.32	0.34	0.46	0.60	0.29
	RP04	0.49	**0.80**	0.51		0.35	0.40	0.50	0.62	0.33
BP	BP01	0.34	0.46	**0.72**		0.47	0.53	0.53	0.37	0.34
	BP02	0.36	0.54	**0.72**		0.44	0.50	0.57	0.43	0.36
GH	GH01	0.24	0.31	0.44		**0.48**	0.46	0.34	0.21	0.26
	GH02	0.23	0.33	0.35		**0.51**	0.39	0.31	0.30	0.29
	GH03	0.08	0.14	0.26		**0.43**	0.28	0.17	0.08	0.11
	GH04	0.12	0.20	0.24		**0.50**	0.31	0.26	0.18	0.23
	GH05	0.22	0.30	0.45		**0.66**	0.50	0.37	0.23	0.31
VT	VT01	0.15	0.23	0.40		0.40	**0.54**	0.32	0.21	0.42
	VT02	0.12	0.20	0.36		0.38	**0.51**	0.30	0.20	0.45
	VT03	0.30	0.41	0.46		0.42	**0.56**	0.44	0.37	0.45
	VT04	0.28	0.38	0.47		0.46	**0.58**	0.43	0.35	0.38
SF	SF01	0.28	0.45	0.56	^a^	0.38	0.45	**0.51**	0.48	0.44
	SF02	0.35	0.47	0.46		0.33	0.41	**0.51**	0.48	0.46
RE	RE01	0.39	0.62	0.40		0.28	0.36	0.54	**0.84**	0.44
	RE02	0.37	0.63	0.39		0.27	0.35	0.52	**0.88**	0.44
	RE03	0.37	0.61	0.39		0.25	0.35	0.49	**0.83**	0.42
MH	MH01	0.26	0.34	0.31		0.23	0.41	0.44	0.43	**0.61**
	MH02	0.27	0.34	0.33		0.29	0.44	0.47	0.44	**0.69**
	MH03	0.09	0.16	0.30		0.28	0.47	0.31	0.23	**0.54**
	MH04	0.22	0.28	0.25		0.23	0.40	0.43	0.42	**0.67**
	MH05	0.05	0.14	0.25		0.28	0.43	0.34	0.24	**0.59**

Numbers in bold show item-own-scale correlations

*PF* Physical function, *RP* Role Physical, *BP* Bodily Pain, *GH* General Health, *VT* Vitality, *SF* Social Function, *RE* Role Emotional, *MH* Mental Health

^a^ Correlation with other scale is higher than correlation with own scale

### Exploratory factor analysis and internal consistency reliability

While all scales were positively correlated, no correlations between scales were strong (above 0.70) supporting the notion that the eight scales measure distinct domains (data not shown). The highest scale correlation (0.67) was seen between RP and RE. In exploratory factor analysis, the first four Eigen values were: 4.07, 1.08, 0.78, 0.57, thus supporting a two-factor solution. In a two-factor model, the factor loadings did not concur with the hypothesized associations (Table 7). Rather, the PF, RP, and RE subscales loaded strongly on first factor (*Physical and role function*), while the BP, GH, VT, SF, and MH subscales loaded strongly on the second factor (*Symptoms, health perception and social function*). Analyses using oblique rotation and analyses of polychoric correlations provided similar results (data not shown). A three factor solution kept the first factor unchanged, but split the second factor into a factor on *Symptoms and general health perception* (BP, GH, and VT loaded strongly on this factor) and a factor on *Social function and mental health* (SF and MH loaded strongly on this factor, which also had a strong cross-loading from RE, data not shown).

**Table 7:** Hypothesized associations and observed factor loadings for a two-factor solution

	Hypothesized associations		Factor loadings Varimax rotation			
	Physical	Mental	Factor1	Factor2	Communality	Alpha
**PF**	●	○	**0.80**	0.07	0.64	0.92
**RP**	●	○	**0.84**	0.28	0.79	0.92
**BP**	●	○	0.43	**0.64**	0.60	0.81
**GH**	◑	◑	0.15	**0.72**	0.55	0.75
**VT**	◑	◑	0.14	**0.85**	0.74	0.75
**SF**	◑	●	0.48	**0.63**	0.62	0.67
**RE**	○	●	**0.74**	0.33	0.65	0.93
**MH**	○	●	0.18	**0.73**	0.56	0.82

*PF* Physical function, *RP* Role Physical, *BP* Bodily Pain, *GH* General Health, *VT* Vitality, *SF* Social Function, *RE* Role Emotional, *MH* Mental Health

●: Strong association (factor loading ≥0.6)

◑ : Moderate association (0.3 < factor loading < 0.6)

○ : Weak association (factor loading ≤0.3)

Bold values indicate factor loadings ≥0.6

Alpha: Cronbach’s alpha

Internal consistency reliability was above the traditional threshold of 0.70 for seven scales. The two-item SF scale had a reliability of 0.67. The internal consistency reliabilities were 0.91 for PCS and 0.90 for MCS.

### Differential item function

We did not identify any DIF with regards to age and sex. Uniform and non-uniform cross-national DIF was identified for 4 and 5 items, respectively, based on explained item variance (Table 8). Due to the large sample size, all DIF results were highly significant. For all 9 items, the direction of DIF was clear and consistent over most or all of the score range. Six items (PF02, *moderate activities*; PF06, *bending/kneeling*; GH01, *health in general*; GH05, *health is excellent*; MH03, *calm and peaceful*; MH05, *happy*) provided a more positive assessment of health in Saudi Arabia compared to the anchor items. Three items (PF10, *bathing or dressing*; GH02, *sick easier*; RE03, *did work less carefully*) provided a more negative assessment of heath in Saudi Arabia compared to the anchor items.

**Table 8:** Test of Differential Item Function (DIF) between Saudi and US SF-36v2 versions

Item	Abbreviated text	dR^2^	DIF	DIF direction for SA
PF01	… vigorous activities, such as running, lifting heavy objects, participating in strenuous sports?	0.021		
PF02	… moderate activities, such as moving a table, pushing a vacuum cleaner, bowling, or playing golf?	**0.045**	**NU**	↑
PF03	… lifting or carrying groceries?	0.020		
PF04	… climbing several flights of stairs?	0.014		
PF05	… climbing one flight of stairs?	0.018		
PF06	… bending, kneeling, or stooping?	**0.121**	**NU**	↑
PF07	… walking more than a mile?	0.027		
PF08	… walking several hundred yards?	0.010		
PF09	… walking one hundred yards?	0.001		
PF10	… bathing or dressing yourself?	**0.035**	**NU**	↓
RP01	cut down on the amount of time	0.010		
RP02	accomplished less than you would like	0.008		
RP03	limited in the kind of work or other activities	0.000		
RP04	difficulty performing the work or other activities	0.000		
BP01	How much bodily pain have you had during the past 4 weeks?	0.005		
BP02	how much did pain interfere with your normal work	0.008		
GH01	In general, would you say your health is:	**0.088**	**NU**	↑
GH02	seem to get sick a little easier than other people	**0.039**	**U**	↓
GH03	as healthy as anybody I know	0.001		
GH04	I expect my health to get worse	0.001		
GH05	health is excellent	**0.058**	**NU**	↑
VT01	did you feel full of life?	0.001		
VT02	did you have a lot of energy?	0.011		
VT03	did you feel worn out?	0.010		
VT04	did you feel tired?	0.003		
SF01	your normal social activities with family, friends, neighbors, or groups?	0.002		
SF02	your social activities (like visiting with friends, relatives, etc.)?	0.003		
RE01	Cut down on the amount of time you spent on work or other activities	0.004		
RE02	Accomplished less than you would like	0.004		
RE03	Did work or other activities less carefully than usual	**0.031**	**U**	↓
MH01	been very nervous?	0.000		
MH02	felt so down in the dumps that nothing could cheer you up?	0.000		
MH03	felt calm and peaceful?	**0.052**	**U**	↑
MH04	felt downhearted and depressed?	0.001		
MH05	been happy?	**0.034**	**U**	↑

dR^2^: Increase in explained item variance by including DIF term, values > 0.03 in bold, NU: non-uniform DIF, U: uniform DIF, ↑: Item score in Saudi Arabia tended to be higher than would be expected from anchor items, ↓: Item score in Saudi Arabia tended to be lower than would be expected from anchor items

### Normative data for Saudi Arabia and comparisons with the USA

Compared to 2009 US general population norms, Saudi Arabia data showed lower scores for the RP, SF, and RE scales as well as for the MCS (Table 9). Slightly lower scores were also seen for the PF and MH scales, but these differences were below the suggested threshold for clinical significance. Adjusting for age and gender led to slightly larger differences for the scales reflecting physical health but had little impact on differences in scales reflecting mental health. Adjusting for DIF lowered the Saudi Arabia scores for PF, GH, MH, PCS and MCS, but provided higher scores for RE, thus slightly diminishing the difference between Saudi Arabia and the US on this scale.

**Table 9:** SF-36v2 Norm Tables for Saudi Arabia – Total Sample

	PF	RP	BP	GH	VT	SF	RE	MH	PCS	MCS
**All**										
Mean	48.73	46.42	50.09	50.58	49.79	46.18	43.18	47.27	50.32	45.36
Std Dev	10.13	9.10	9.33	8.51	9.20	8.90	11.21	10.15	8.12	9.69
Minimum	19.26	21.23	21.68	18.95	22.89	17.23	14.39	11.63	16.40	7.74
25th Pctl	44.15	39.19	42.64	46.05	43.69	42.30	35.28	40.40	44.82	39.17
50th Pctl	51.80	48.17	51.51	50.81	49.63	47.31	45.72	48.25	51.55	46.25
75th Pctl	57.54	54.91	55.55	56.99	55.57	52.33	56.17	56.10	56.72	52.47
Maximum	57.54	57.16	62.00	66.50	70.42	57.34	56.17	63.95	72.45	73.18
N	6164	6148	6150	6166	6149	6163	6135	6150	6136	6137
N Miss	2	18	16	0	17	3	31	16	30	29
δ-USA unadj (95% CI)	−1.27 (−1.67/ -0.87)	−3.58 (−3.96/ -3.20)	0.09 (−0.29/ 0.47)	0.58 (0.22/ 0.94)	−0.21 (−0.59/ 0.16)	−3.82 (−4.20/ -3.45)	−6.82 (−7.25/ -6.40)	−2.73 (− 3.14/ -2.33)	0.31 (− 0.04/ 0.66)	−4.65 (− 5.04/ -4.26)
δ-USA adj (95% CI)	−2.70 (−3.11/ -2.28)	**−4.75** (− 5.15/ -4.36)	**− 0.96** (− 1.36/ -0.56)	0.04 (− 0.35/ 0.43)	**−0.25** (− 0.65/ 0.15)	**− 4.23** (− 4.63/ -3.84)	− 7.15 (− 7.60/ -6.69)	− 2.44 (− 2.87/ -2.01)	− 1.18 (− 1.55/ -0.82)	−4.18 (− 4.59/ -3.76)
δ-USA DIF adj (95% CI)	**− 3.10** (− 3.51/− 2.70)			**−1.83** (−2.20/−1.47)			**− 5.67** (− 6.10/− 5.23)	**−4.82** (−5.25/− 4.38)	**−1.58** (−1.94/− 1.22)	**− 4.57** (− 4.98/− 4.16)

Figures in **BOLD** indicate our best assessment of crossnational differences

δ-USA unadj: Difference between Saudi and US SF-36v2 scores without adjustment

δ-USA adj: Difference between Saudi and US SF-36v2 scores with adjustment for differences in age and gender

δ-USA DIF adj: Difference between Saudi and US SF-36v2 scores with adjustment for differential item function and differences in age and gender

*95% CI* 95% confidence interval, *PF* Physical function, *RP* Role Physical, *BP* Bodily Pain, *GH* General Health, *VT* Vitality, *SF* Social Function, *RE* Role Emotional, *MH* Mental Health

Both in Saudi Arabia and in the USA, separate analyses by gender and age group (Tables 10 and 11) showed lower physical health scores for older age groups. However, this trend was most pronounced in the USA, so the strongest cross-national differences were seen in the younger age groups. Saudi Arabian women, 60 years or older, reported significantly better physical function than American women in the same age group.

**Table 10:** SF-36v2 Norm Tables for Saudi Arabia – Age Groups – Male

	PF	RP	BP	GH	VT	SF	RE	MH	PCS	MCS
**15–29 Years**										
Mean	49.94	47.33	51.99	51.62	51.25	47.29	45.22	47.96	51.49	46.62
Std Dev	10.39	9.60	9.61	8.68	8.75	9.03	11.61	9.80	8.07	9.27
Minimum	19.26	21.23	21.68	18.95	22.89	17.23	14.39	11.63	22.82	11.46
25th Pctl	46.06	39.19	46.27	46.05	46.66	42.30	35.28	40.40	46.17	40.67
50th Pctl	55.63	48.17	51.51	52.23	52.60	47.31	45.72	50.87	52.99	47.69
75th Pctl	57.54	57.16	62.00	57.94	58.54	57.34	56.17	56.10	57.93	53.15
Maximum	57.54	57.16	62.00	66.50	70.42	57.34	56.17	63.95	68.30	69.98
N	1265	1263	1264	1266	1264	1265	1260	1264	1262	1263
N Miss	1	3	2	0	2	1	6	2	4	3
δ-USA unadj (95% CI)	−5.63 (−6.73/ -4.52)	−7.23 (−8.28/− 6.19)	− 2.93 (− 4.01/ -1.86)	−2.19 (− 3.21/ -1.17)	−0.99 (− 2.03/ 0.04)	−4.72 (− 5.75/ -3.68)	−7.58 (−8.85/ -6.32)	− 3.07 (− 4.20/ -1.95)	− 4.28 (− 5.17/ -3.39)	− 3.76 (− 4.82/ -2.70)
δ-USA DIF adj (95% CI)	− 5.96 (−7.03/−4.89)			− 4.15 (−5.10/−3.20)			−6.38 (−7.56/−5.19)	−5.42 (−6.58/−4.27)	−4.62 (− 5.50/− 3.75)	− 4.28 (−5.32/− 3.23)
**30–44 Years**										
Mean	48.45	46.41	50.84	51.91	51.37	46.56	44.08	48.10	50.47	46.63
Std Dev	10.74	9.15	9.39	8.43	8.89	8.92	11.21	9.48	8.13	9.37
Minimum	19.26	21.23	21.68	18.95	22.89	17.23	14.39	11.63	22.82	8.57
25th Pctl	42.23	39.19	42.64	47.48	46.66	42.30	35.28	43.02	44.81	40.66
50th Pctl	53.71	48.17	51.51	53.19	52.60	47.31	45.72	50.87	51.80	47.22
75th Pctl	57.54	54.91	62.00	57.94	58.54	52.33	56.17	56.10	56.89	53.46
Maximum	57.54	57.16	62.00	66.50	70.42	57.34	56.17	63.95	72.45	69.84
N	804	801	801	804	801	804	799	802	799	799
N Miss	0	3	3	0	3	0	5	2	5	5
δ-USA unadj (95% CI)	−5.10 (−6.17/ -4.03)	−6.47 (−7.46/−5.49)	−1.51 (− 2.56/ -0.46)	0.31 (− 0.66/ 1.29)	−0.13 (− 1.15/ 0.90)	−5.15 (− 6.17/ -4.13)	−7.32 (−8.51/ -6.13)	−2.02 (− 3.13/ -0.91)	−2.93 (− 3.79/ -2.06)	−3.42 (−4.51/ -2.33)
δ-USA DIF adj (95% CI)	− 5.42 (− 6.46/−4.39)			− 1.71 (−2.63/−0.78)			− 5.96 (−7.09/− 4.83)	−4.35 (−5.48/− 3.22)	−3.31 (− 4.17/− 2.46)	−3.86 (−4.94/− 2.78)
**45–59 Years**										
Mean	48.42	46.54	50.83	50.73	50.97	46.49	43.85	47.67	50.29	46.19
Std Dev	10.58	9.27	9.07	8.80	8.61	8.52	11.42	9.43	7.58	9.10
Minimum	19.26	21.23	21.68	18.95	22.89	17.23	14.39	14.24	22.82	7.74
25th Pctl	44.15	39.19	46.27	46.05	46.66	42.30	35.28	40.40	45.35	40.38
50th Pctl	51.80	48.17	51.51	50.81	49.63	47.31	45.72	48.25	51.09	46.99
75th Pctl	57.54	57.16	62.00	56.99	58.54	52.33	56.17	56.10	56.18	52.87
Maximum	57.54	57.16	62.00	66.50	70.42	57.34	56.17	63.95	68.30	73.18
N	514	514	512	514	514	514	511	514	514	514
N Miss	0	0	2	0	0	0	3	0	0	0
δ-USA unadj (95% CI)	−1.67 (−2.87/ -0.46)	−2.65 (−3.84/−1.47)	2.17 (0.99/ 3.35)	2 (0.87/ 3.12)	−0.05 (−1.16/ 1.05)	−3.03 (−4.18/ -1.88)	−6.11 (−7.38/ -4.84)	−2.56 (− 3.75/ -1.37)	1.31 (0.24/ 2.37)	−4.28 (−5.44/ -3.13)
δ-USA DIF adj (95% CI)	−2.05 (−3.23/−0.88)			0.11 (− 0.97/1.19)			−4.74 (−5.97/− 3.52)	−4.93 (−6.14/− 3.72)	0.94 (−0.12/2.00)	−4.73 (−5.88/− 3.58)
**60+ Years**										
Mean	46.63	45.39	49.64	50.49	51.37	45.55	44.49	48.72	48.43	47.42
Std Dev	11.65	9.35	9.81	9.42	9.25	9.56	11.01	10.25	8.67	9.61
Minimum	19.26	21.23	25.71	18.95	22.89	17.23	14.39	11.63	25.20	16.54
25th Pctl	40.32	39.19	42.24	43.68	46.66	37.29	35.28	40.40	41.29	40.92
50th Pctl	49.88	45.93	51.51	52.23	52.60	47.31	45.72	50.87	50.20	48.43
75th Pctl	55.63	54.91	55.55	57.94	58.54	52.33	56.17	58.72	55.38	55.15
Maximum	57.54	57.16	62.00	66.50	70.42	57.34	56.17	63.95	64.78	64.48
N	216	213	214	216	215	216	214	215	213	213
N Miss	0	3	2	0	1	0	2	1	3	3
δ-USA unadj (95% CI)	0.52 (−0.95/ 2.00)	−1.04 (−2.44/ 0.36)	1.09 (−0.23/ 2.42)	1.81 (0.49/ 3.13)	0.14 (− 1.17/ 1.45)	−5.12 (−6.42/ -3.83)	−6.03 (−7.35/ -4.72)	−4.65 (− 5.86/ -3.44)	2.92 (1.54/ 4.29)	−6.41 (−7.60/ -5.21)
δ-USA DIF adj (95% CI)	0.25 (−1.19/1.70)			−0.01 (− 1.29/1.26)			−4.69 (−5.96/−3.42)	−6.97 (−8.19/− 5.74)	2.61 (1.24/3.97)	−6.86 (−8.04/− 5.67)

δ-USA unadj: Difference between Saudi and US SF-36v2 scores without adjustment

δ-USA DIF adj: Difference between Saudi and US SF-36v2 scores with adjustment for differential item function

*95% CI* 95% confidence interval, *PF* Physical function, *RP* Role Physical, *BP* Bodily Pain, *GH* General Health, *VT* Vitality, *SF* Social Function, *RE* Role Emotional, *MH* Mental Health

**Table 11:** SF-36v2 Norm Tables for Saudi Arabia – Age Groups – Female

	PF	RP	BP	GH	VT	SF	RE	MH	PCS	MCS
**15–29 Years**										
Mean	49.13	46.70	49.78	50.16	48.98	46.20	42.17	46.99	50.60	44.52
Std Dev	9.53	8.61	8.90	8.20	9.28	8.62	10.98	10.36	7.91	9.82
Minimum	19.26	21.23	21.68	18.95	22.89	17.23	14.39	11.63	16.89	8.33
25th Pctl	46.06	39.19	42.64	46.05	43.69	42.30	35.28	40.40	45.04	38.02
50th Pctl	51.80	48.17	50.71	50.81	49.63	47.31	45.72	48.25	51.96	45.30
75th Pctl	55.63	54.91	55.55	55.56	55.57	52.33	52.69	56.10	56.88	51.89
Maximum	57.54	57.16	62.00	66.50	70.42	57.34	56.17	63.95	69.64	66.24
N	1678	1672	1673	1678	1675	1677	1670	1675	1671	1671
N Miss	0	6	5	0	3	1	8	3	7	7
δ-USA unadj (95% CI)	−3.78 (−4.73/ -2.83)	−5.65 (−6.52/−4.78)	−2.75 (− 3.67/ -1.84)	−0.44 (−1.31/ 0.43)	0.93 (− 0.05/ 1.90)	−3.51 (−4.43/ -2.59)	−7.02 (−8.13/− 5.90)	−0.71 (−1.78/ 0.35)	−3.07 (− 3.87/ -2.27)	−2.26 (−3.30/− 1.22)
δ-USA DIF adj (95% CI)	− 4.22 (− 5.13/− 3.31)			− 2.29 (−3.10/−1.49)			− 5.44 (−6.49/−4.38)	−3.13 (− 4.23/− 2.03)	−3.49 (−4.28/− 2.70)	− 2.62 (−3.65/− 1.59)
**30–44 Years**										
Mean	48.36	45.99	48.59	49.38	47.88	45.12	41.48	46.10	49.77	43.61
Std Dev	9.41	8.70	8.94	8.44	9.30	8.99	10.90	10.63	8.01	10.04
Minimum	19.26	21.23	21.68	18.95	22.89	17.23	14.39	11.63	17.76	11.10
25th Pctl	44.15	39.19	42.24	43.68	43.69	37.29	35.28	37.79	44.91	37.05
50th Pctl	51.80	48.17	46.68	49.86	46.66	47.31	42.24	45.64	50.77	44.39
75th Pctl	55.63	52.66	55.55	55.56	55.57	52.33	49.20	55.23	55.87	51.23
Maximum	57.54	57.16	62.00	66.50	70.42	57.34	56.17	63.95	69.57	64.75
N	880	879	881	881	878	881	878	878	876	876
N Miss	1	2	0	0	3	0	3	3	5	5
δ-USA unadj (95% CI)	−3.65 (−4.67/ -2.63)	−5.84 (−6.81/− 4.86)	−2.31 (− 3.33/ -1.29)	−1.01 (− 2.02/ -0.01)	−0.75 (− 1.80/ 0.31)	−4.73 (− 5.78/ -3.69)	−8.11 (−9.30/−6.92)	−2.72 (− 3.86/ -1.58)	−2.41 (− 3.32/ -1.49)	−4.49 (−5.61/− 3.36)
δ-USA DIF adj (95% CI)	− 4.17 (−5.16/− 3.18)			− 2.78 (− 3.73/− 1.84)			−6.46 (−7.60/− 5.31)	−5.16 (− 6.33/− 3.99)	− 2.85 (− 3.75/− 1.95)	− 4.80 (− 5.93/− 3.68)
**45–59 Years**										
Mean	47.89	45.42	48.48	49.81	48.66	45.00	41.99	46.74	49.22	44.51
Std Dev	9.28	8.81	9.38	8.21	9.58	8.85	10.35	10.12	8.14	9.37
Minimum	19.26	21.23	21.68	23.71	22.89	17.23	14.39	11.63	21.40	14.76
25th Pctl	42.23	39.19	42.24	44.31	43.69	37.29	35.28	39.10	43.68	39.03
50th Pctl	51.80	45.93	50.71	50.81	46.66	47.31	45.72	48.25	50.49	45.31
75th Pctl	55.63	52.66	55.55	55.56	55.57	52.33	49.20	53.48	55.78	51.49
Maximum	57.54	57.16	62.00	66.50	70.42	57.34	56.17	63.95	68.07	64.24
N	429	429	429	429	428	429	427	428	428	428
N Miss	0	0	0	0	1	0	2	1	1	1
δ-USA unadj (95% CI)	0.85 (−0.46/ 2.16)	−2.42 (−3.74/ -1.10)	1.88 (0.56/ 3.20)	1.74 (0.45/ 3.03)	0.55 (− 0.79/ 1.89)	−3.2 (−4.51/ -1.88)	−6.22 (−7.66/ -4.77)	−1.46 (−2.84/ -0.07)	2.06 (0.81/ 3.31)	−4.23 (−5.58/− 2.89)
δ-USA DIF adj (95% CI)	0.37 (−0.92/1.65)			−0.11 (−1.36/1.14)			−4.57 (− 5.98/−3.16)	−3.84 (− 5.24/−2.43)	1.61 (0.36/2.85)	−4.54 (− 5.88/− 3.19)
**60+ Years**										
Mean	46.27	42.98	46.12	48.79	46.66	43.88	40.57	45.97	47.08	43.64
Std Dev	10.76	10.11	9.80	8.90	9.78	9.75	10.95	10.76	10.02	10.53
Minimum	19.26	21.23	21.68	26.08	22.89	17.23	14.39	11.63	16.40	11.42
25th Pctl	41.00	36.95	38.21	43.68	40.72	37.29	35.28	37.79	42.53	36.92
50th Pctl	48.93	41.44	46.68	50.81	46.66	42.30	38.76	48.25	47.99	42.85
75th Pctl	55.63	52.66	51.51	55.09	52.60	52.33	45.72	53.48	54.25	50.88
Maximum	57.54	57.16	62.00	66.50	67.45	57.34	56.17	63.95	65.20	66.07
N	168	167	168	168	168	168	167	168	167	167
N Miss	0	1	0	0	0	0	1	0	1	1
δ-USA unadj (95% CI)	3.18 (1.42/ 4.94)	−2.27 (−3.97/−0.57)	−0.54 (−2.09/ 1.01)	−0.17 (−1.72/ 1.38)	−3.2 (−4.76/−1.64)	−5.16 (−6.72/ -3.60)	−8.54 (−10.17/−6.91)	−5.59 (−7.10/ -4.07)	3.17 (1.47/ 4.86)	−8.94 (−10.48/−7.41)
δ-USA DIF adj (95% CI)	2.76 (1.03/4.50)			−1.92 (−3.43/−0.41)			−6.84 (−8.44/−5.23)	−7.99 (−9.52/−6.46)	2.75 (1.07/4.43)	−9.25 (− 10.77/− 7.72)

δ-USA unadj: Difference between Saudi and US SF-36v2 scores without adjustment

δ-USA DIF adj: Difference between Saudi and US SF-36v2 scores with adjustment for differential item function

*95% CI* 95% confidence interval, *PF* Physical function, *RP* Role Physical, *BP* Bodily Pain, *GH* General Health, *VT* Vitality, *SF* Social Function, *RE* Role Emotional, *MH* Mental Health

Comparisons according to gender in the Saudi Arabian sample showed that women scored lower on several scales: BP, GH, VT, SF, RE, and MH as well as on MCS (Tables 10 and 11). However, except for VT, the score differences were below the thresholds for clinical significance.

Among men in Saudi Arabia (Table 10), the strongest score differences across age groups were seen for the PF scale and PCS. Among women (Table 11), lower scores in older age groups were seen for the RP, BP, and VT scales and PCS, whereas other scales remained fairly constant across age.

## Discussion

This nationwide study generally supported the content validity, construct validity, and reliability of a new Saudi version of the SF-36v2. In the qualitative study, participants emphasized physical and psychological function as important components of health – along with social function and healthy eating. Thus, similar to the World Health Organization (WHO) definition [23], health was seen as having both physical, psychological and social aspects. Most of the important HRQOL outcomes listed by participants overlap with domains covered by SF-36. However, some domains mentioned by smaller proportions of participants are not covered by the SF-36: religious habits, eating habits, travel, good sleep, and sexual function. Items covering these domains have been developed and will be reported in future papers.

Cognitive debriefing of the Saudi SF-36v2 indicated that respondents found the questionnaire easy to understand and answer. Each survey item was rated as relevant by more than 90% of participants, supporting the content validity of the survey.

The psychometric analyses supported the reliability and validity of the SF-36v2 in a Saudi general population. All items showed satisfactory convergence within scales. In all but one instance, items also showed satisfactory differentiation across scales. Such results are on par with results from the original validation of the SF-36 in the US [29]. Overall, these results support the hypothesized scale structure of the Saudi Arabic SF-36v2. However, exploratory factor analyses did not find a factor solution similar to typical results from Western countries [31]. Rather, the two-factor solution resembled results previously found in a Japanese sample [32] and to some extent in a Turkish urban population [21]. In contrast, factor analytic results from a study in Lebanon more closely resembled typical results from western countries [7]. The factor solution in our study seems particularly driven by the high correlation between the RP and RE scales, which suggest that the distinction between physical and psychological reasons for poor role performance does not apply to the Saudi data. The implications of these results for the validity of the PCS and MCS scores in Saudi Arabia needs to be explored in future studies.

Seven of the SF-36v2 scales had internal consistency reliability above 0.70, but the two-item SF scale had a reliability of only 0.67. However, this scale has also shown low reliability in some US studies, e.g., the first US general population study, where the SF scales showed a reliability of 0.63 [41]. Thus, the reliability results may be considered as adequate.

Within Saudi Arabia, we found no DIF for age and gender, but we found cross-national DIF for 9 items when comparing with US general population data. In a post-hoc cognitive debriefing study of these 9 items we were not able to identify problems in these 9 items that might explain the DIF (data not shown). A possible explanation of the DIF may be cultural or lifestyle differences between Saudi Arabia and the USA. For example, because of religious practices, persons in Saudi Arabia may do more bending and kneeling and thus find this activity easier than persons in the USA. The item on bending and kneeling is still a valid indicator of physical function in each country, but the item is easier for persons in Saudi Arabia, thus influencing comparisons of Physical Function. If the interest of the researcher is to compare physical function in general (and not the specific activity of bending/kneeling) the comparison can be adjusted for the DIF. The impact of such adjustments can be evaluated on the overall level in Table 9 and for age and gender subgroups in Tables 10 and 11. The impact is actually rather small for the PF scale (0.40), but larger for the GH (1.87) and MH (2.38) scales. While these impacts are smaller than the MID for each scale, the largest impacts are larger than the impact of adjustment for demographic differences. Therefore, we recommend considering DIF when interpreting cross-national comparisons between Saudi Arabia and the USA.

After adjustment for differences in age and gender, as well as DIF, analysis of Saudi general population norm data showed low scores for scales concerning physical function (PF difference = − 3.10), role and social function (RP difference = − 4.75, SF difference = − 4.23, and RE difference = − 5.67), mental health (MH difference = − 4.82) as well as for the mental component summary (MCS difference = − 4.57) compared to US general population norms (Table 9). In particular, scores on the RE scale were lower for women in Saudi Arabia compared to the USA, although some of this difference was explained by DIF. These differences are not likely to be caused by higher morbidity in Saudi Arabia since the self-reported prevalence of many chronic conditions was lower in Saudi Arabia than in the USA. The magnitude of the differences on these scales suggests differences in function that need to be explored. In particular, the lower scores in scales relating to mental health (SF, RE, MH, and MCS) does not concur with the low reports of clinical anxiety and depression (Table 5). A large study (1990–2013) to estimate the burden of mental disorders in the Eastern Mediterranean Region including Saudi Arabia, reported that the stigma attached to mental illness may cause underreporting or waiting for a long period of time before seeking healthcare [42]. Thus, it is possible that clinical anxiety and depression is under-diagnosed or under-reported for cultural reasons. Further, the low score on scales related to mental health may reflect subclinical, rather than clinical, mental health problems.

As in previous general population studies (e.g. [3, 4, 9, 10]), women scored lower on all SF-36v2 scales, thus supporting known groups validity. However, the average differences were often small – only the gender difference for the vitality scale exceeded the threshold for clinical significance.

Analyses by age group found lower scores in older age groups for SF-36v2 scales concerning physical health: PF, RP, BP, and PCS. These results are in line with results from many other studies [3, 4, 9, 10], reflecting a decline in physical function with age and thus supporting known groups validity. As in previous studies, measures reflecting mental health were relatively constant across age groups. A study by Lorem et al. [43] found age by itself was protective of mental health symptoms when controlled for the mental health symptoms associated with physical illness.

Representation from most regions of Saudi Arabia was satisfactory, but few participants were recruited from the Northern region. We ascribe these difficulties in recruiting participants to lack of familiarity and lack of acceptance of surveys in some parts of the Saudi culture. The Northern region is the smallest (285,733 Saudi inhabitants in 2016) and least densely populated region in Saudi Arabia, with a population that is slightly younger (mean age 26.1 years against 27.4 years for all of Saudi Arabia) and with a slightly lower proportion of mean (50,3% against 50.9%). However, since these differences are very small, the low proportion of participants from the Northern region is unlikely to have a noticeable impact on the overall results.

## Conclusion

This is the first large scale Saudi general population study of patient reported outcomes. We used a new translation of a well-known patient reported outcomes instrument, the SF-36v2. Concept elicitation, cognitive debriefing, and large-scale quantitative testing supported the validity and reliability of the Saudi SF-36v2, but an exploratory factor analysis did not support the typical distinction between a physical health and a mental health component. Also, we found cross-national DIF for 9 out of 35 tested items. After adjustment for DIF and demographic differences we found lower patient reported outcomes scores in Saudi Arabia for the PF, RP, SF, RE and MH scales as well as for the MCS. For the BP, GH and VT scales, as well as for PCS, score differences were smaller and did not exceed MID. Reasons for the differences in patient reported outcomes should be further explored and these general population differences should be taken into account when interpreting patient reported outcomes scores for patients in Saudi Arabia.

## Data Availability

The datasets/tables used and/or analyzed during the current study are available from the author on reasonable request.

## Abbreviations

HRQOL: Health-related Quality of Life
IQOLA: International Quality of Life Assessment
SF-36v2: Short Form Health Survey
DIF: Differential item function
PRO: Patient-reported outcome
IRT: Item response theory
MID: Minimally important difference
WHO: World Health Organization
MCS: The Mental Component Summary
PCS: The Physical Component Summary
MH: Mental Health
RE: Role limitations due to Emotional Problems
SF: Social Function
VT: Vitality
GH: General Health Perception
BP: Bodily Pain
RP: Role Limitations due to Physical Health
PF: Physical Function
US: United States
